# Identification of New Key Players for Ferrous Iron Export in the Asymmetric Inner Gate of Human Ferroportin 1

**DOI:** 10.1096/fj.202500790RR

**Published:** 2025-07-10

**Authors:** Marlène Le Tertre, Ahmad Elbahnsi, Cécile Ged, Kevin Uguen, Isabelle Gourlaouen, Claude Férec, Chandran Ka, Gérald Le Gac, Isabelle Callebaut

**Affiliations:** ^1^ Univ Brest, Inserm, EFS Brest France; ^2^ CHU de Brest Brest France; ^3^ Sorbonne Université, Muséum National d'Histoire Naturelle, UMR CNRS 7590, Institut de Minéralogie, de Physique des Matériaux et de Cosmochimie, IMPMC Paris France; ^4^ Université Paris Cité, CNRS UMR 8038 CiTCoM, Inserm U1268 MCTR Paris France; ^5^ Service de Biochimie‐Analyses Génétiques, CHU de Bordeaux, Inserm U1035, Biothérapie des Maladies Génétiques, Inflammatoires et Cancers, Université de Bordeaux Bordeaux France; ^6^ CHU Guadeloupe, Laboratoire de Génétique Moléculaire Pointe à Pitre France; ^7^ Association Gaétan Saleün Brest France; ^8^ Laboratory of Excellence GR‐Ex Paris France

**Keywords:** ferroportin disease, human FPN1, intracellular gate, iron metabolism, MFS transporters

## Abstract

The Major Facilitator Superfamily (MFS) is the largest known family of secondary transporters. These proteins share a common architecture comprising two lobes, each including 6 transmembrane (TM) helices, related by twofold pseudosymmetry. They transport a wide range of substrates through large conformational changes relying on the opening and closing of gates located on either side of biological membranes. Human ferroportin 1 (HsFPN1), the sole characterized mammalian iron exporter, follows this pattern. It is, however, characterized by an unusual intracellular gate, formed by two asymmetric networks of non‐covalent bonds linking the two lobes. We studied the behavior of these networks in all‐atom molecular dynamics simulations and functionally assessed the effect of alanine substitutions on HsFPN1 plasma membrane expression and iron export activity. We identified two new critical residues, Arg156 and Tyr318, connecting the networks to each other and to one of two metal‐coordinating sites, located in an unwound region of TM7. We extended the analysis to a previously unreported missense variation, p.Gln478Arg, which was found to have a very strong impact on one of the two inter‐lobe connection networks, and to result in a significant HsFPN1 loss‐of‐function. This led us to present the p.Gln478Arg substitution as a new pathogenic variation causing ferroportin disease. Together, our results provide new insights into the structure and dynamics of the human FPN1 inner gate and its asymmetry, shedding light on its potential role in the mechanism of iron export while offering a framework to better understand previously unexplained clinical observations.

## Graphical abstract

Ferroportin 1 is an iron exporter belonging to the Major Facilitator Superfamily. Its unusual intracellular gate is formed by asymmetric networks of non‐covalent bonds. We analyzed these networks using Molecular Dynamics simulations and experimental tests and identified novel amino acids as key players in inter‐lobe connections. This work deepens our understanding of the molecular mechanisms involved in iron transport and sheds light on clinical observations.

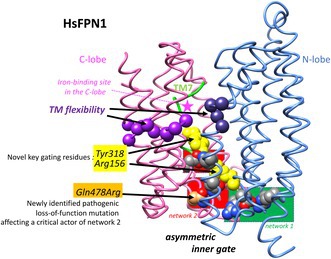

**Abbreviations**
CHOL: cholesterol
FPN1: ferroportin 1
IF: inward‐facing
MD: Molecular Dynamics
MFS: Major Facilitator Superfamily
OF: Outward‐facing
POPC: 1‐palmitoyl‐2‐oleoyl‐sn‐glycero‐3‐phosphocholine
POPE: 1‐palmitoyl‐2‐oleoyl‐sn‐glycero‐3‐phosphoethanolamine
POPS: 1‐palmitoyl‐2‐oleoyl‐sn‐glycero‐3‐phospho‐L‐serine
TM: TransMembrane
WT: Wild‐Type

## 1 Introduction

Iron is an essential element for almost all living organisms, but it is also toxic at high levels due to its propensity to change oxidation state and cause the degradation of numerous biomolecules (e.g., lipids, DNA, proteins). In mammals, different mechanisms have emerged to adjust cellular and serum iron levels to the body's requirements [1]. Hepcidin, a circulating peptide of 25 amino acids mainly synthesized by the liver and secreted into plasma [2], is the master regulator of systemic iron homeostasis [3]. It acts by reducing the activity of ferroportin 1 (FPN1 or SLC40A1, Uniprot #Q9NP59), the only known exporter of cellular iron, in the plasma membrane of a variety of cells, primarily macrophages, duodenal enterocytes, and hepatocytes [1, 4, 5, 6, 7].

FPN1 is a member of the major facilitator superfamily (MFS), including secondary active transporters that control the flow of a wide variety of molecules (inorganic ions, metabolites, neurotransmitters, toxins, drugs, and other substrates) across biological membranes [8, 9]. The three‐dimensional (3D) structure of MFS members consists of two bundles of six transmembrane helices (N‐lobe: TM1‐TM6; C‐lobe: TM7‐TM12) that interact together and orchestrate transitions between two extreme inward‐facing (IF) and outward‐facing (OF) open conformations. During the conformational transitions, the substrate is located in a central cavity and is not accessible to either side of the membrane [8, 10, 11, 12].

How the MFS conformational changes are achieved is a central question. Several studies have highlighted the role of “gating residues”, which mediate non‐covalent interactions (salt bridges and hydrogen bonds) between the N‐ and C‐lobes. These interactions are alternatively formed and broken along the MFS transport cycle [8, 11, 13]. The A‐motif, which is characteristic of the MFS superfamily and is invariably found between TM2 and TM3 in the N lobe and/or between TM8 and TM9 in the C lobe, plays a key role in the closure of the transporters at the inner side of the plasma membrane. More specifically, a basic residue of the A‐motif on TM3 or TM9 forms an intra‐lobe salt bridge with an acidic residue on TM4 or TM10, while this latter is also bridged to a basic residue on TM11 or TM5, thereby contributing to the formation of an intracellular barrier, or inner gate [11].

**FIGURE 1:**
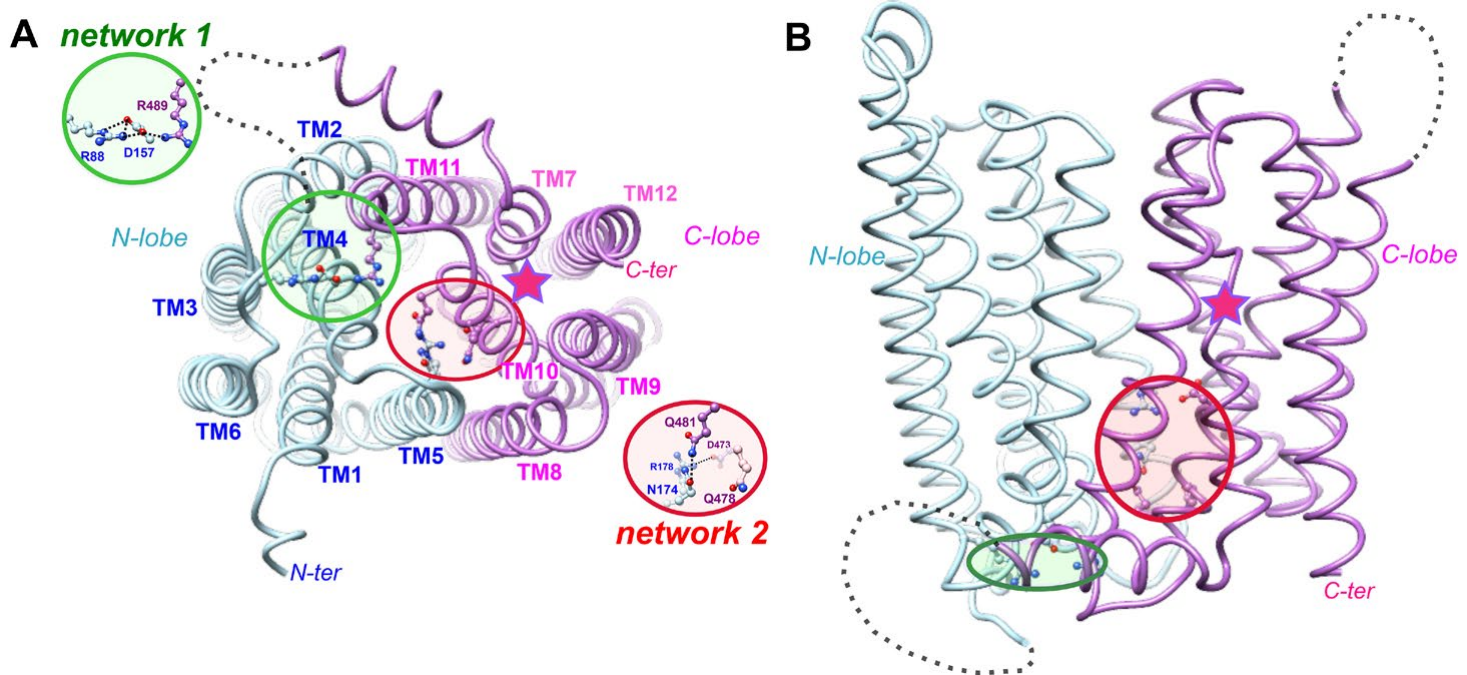
Networks of non‐covalent bonds in the intracellular gate of HsFPN1. Ribbon representation of the experimental 3D structure of human ferroportin 1 (HsFPN1) in the OF state (pdb 6W4S). A and B correspond to views from the cytosol and in the membrane plane (lateral view), respectively. The N and C‐lobes are depicted in blue and pink, respectively, and their TM helices are labeled and colored accordingly. Missing loops are depicted with dashed lines. Amino acids of the two networks are shown in the green (network 1) and red (network 2) circles (the details are shown on the left and right in panel A). The unwound segment of TM7 is highlighted with a star. Amino acid names are indicated using the one‐letter code. Gln(Q)478 is shown in addition to the amino acids observed in the cryo‐EM 3D structure as participating in network 2. Indeed, this residue made a stable H‐bond with Asn(Q)174 in MD simulations we previously made with a 3D structure model of HsFPN1 [14].

The specific features of the human ferroportin 1 (HsFPN1) inner gate bond networks were first highlighted in a pioneering study we performed [14] that combined in vitro analyses and short molecular dynamics (MD) simulations on a model of the HsFPN1 3D structure built using the crystal structure of *Bdellovibrio* BbFpn1 as a template [15]. They were subsequently confirmed in the experimental 3D structure of HsFPN1 solved in an apo, OF state by cryo‐electron microscopy (cryo‐EM) [16]. The A‐motif has been identified in the N‐lobe, involving Arg88 (TM3), which forms a salt bridge with Asp157 (TM4), itself bridged to Arg489 (TM11) (network 1 in green in Figure 1). It is the central point of a larger network of charged/polar amino acids, also involving Asp84 (TM2) and Glu486 (TM11) [14, 16, 17]. The two Arg88‐Asp157 and Asp157‐Arg489 interacting pairs are conserved in the primate tarsier protein TsFPN1 (Arg88‐Asp157 and Asp157‐Arg490), as well as in the bacterial homolog BbFpn1 (Arg73‐Asp140 and Asp140‐Arg371) [15, 18] (Figure S1). The A‐motif is, by contrast, absent from the C‐lobe, where inter‐lobe interactions are mediated by two rungs of non‐covalent bonds (network 2 in red in Figure 1), which are positioned deeper in the bilayer, further away from the membrane‐cytosol interface [14, 16] (Figure 1B). This network also shows differences between HsFPN1 and BbFpn1 (Figure S1) and includes in HsFPN1 a salt bridge between Arg178 (TM5) and Asp473 (TM10), and hydrogen bonds between Asn174 (TM5) and Gln481 (TM10).

The aim of this study was to explore in greater depth the asymmetric inner gate of HsFPN1 by investigating the behavior of the two charged/polar residue networks in a complex lipid environment using all‐atom molecular dynamics (MD) simulations performed on the HsFPN1 cryo‐EM 3D structure. This theoretical analysis was combined with site‐directed mutagenesis and biochemical assays to reveal new critical players of the inner gate. These link the two bond networks together and connect them to a non‐helical stretch interrupting helix TM7, a structural feature considered critical for the binding of iron and the iron‐regulatory peptide hepcidin [15, 16, 19]. Our study also confirmed the critical role of Gln478, which is otherwise the site of a missense variation (p.Gln478Arg) leading to an iron overload phenotype. As a whole, this study reveals a series of structural features accounting for the atypical asymmetric behavior of ferroportin within the Major Facilitator Superfamily.

## 2 Material and Methods

### 2.1 Molecular Dynamics (MD) Simulations

HsFPN1 cryo‐EM 3D structure in an apo, outward‐facing (OF) state (PDB ID 6W4S) [16] was prepared using the CHARMM‐GUI server [20, 21, 22, 23]. Simulations were conducted for both the wild‐type (WT) and the Gln(Q)478Arg(R)‐mutated protein. Due to their considerable lengths, missing loops within the HsFPN1 structure were not reconstructed. This decision was made to maintain structural integrity and avoid speculative modeling.

The HsFPN1 protein was embedded in a lipid bilayer composed of POPC, POPE, POPS (1‐palmitoyl‐2‐oleoyl‐sn‐glycero‐3‐phospho‐L‐serine), and CHOL in a 2:1:1:1 ratio. The position of the bilayer was estimated thanks to the PPM server [24, 25]. All systems were solvated using a solution of 150 mM NaCl + CaCl2. The protein, lipids, and ions were modeled using the CHARMM36m force field [26, 27] and the TIP3P model was employed for water. The calculations involved a series of steps, including minimization, equilibration, and production runs, which were executed using different versions of Gromacs (2019, 2020 and 2023) [28, 29] depending on the specific computer or server utilized. During the minimization and equilibration steps, the standard CHARMM‐GUI inputs were used. Harmonic restraints were initially placed on the heavy atoms of the protein and the lipid heads, with a gradual release over a period of 1.2 ns.

The production dynamics were conducted in the NPT ensemble, without any restraints, and temperature and pressure were maintained at 310 K and 1 bar, respectively, using the Nose–Hoover thermostat [30] and Parrinello–Rahman barostat [31]. Periodic boundary conditions were applied, and long‐range electrostatic interactions were treated using the particle mesh Ewald algorithm [32], with a switching function employed between 10 and 12 Å for non‐bonded interactions. Bond lengths involving hydrogen atoms were constrained using LINCS [33]. The integration timestep was set at 2 fs, and the total trajectory length was ~1000 ns.

For the analysis of MD simulations, Root Mean Square Deviation (RMSD) and Root Mean Square Fluctuation (RMSF) calculations for the protein were carried out using the Bio3D module in R [34]. Both RMSD and RMSF analyses were conducted on the Cα atoms. Secondary structure calculations of the TM helices were performed using the *cpptraj* module [35] of AmberTools2024 with the secstruct function based on the DSSP algorithm. The RMSD, RMSF, and secondary structure figures were plotted with gnuplot Version 5.2 [36].

### 2.2 Contact Analysis

The contacts of amino acids (aa) of interest with their environment (including other amino acids and lipids) were calculated with the Voronoi Laguerre Delaunay for Macromolecules (VLDM) program [37, 38]. VLDM relies on a tessellation method, that is, a partition of space into a collection of polyhedra filling space without overlaps or gaps. Delaunay tessellation and its Laguerre dual are performed using a set of heavy‐atom cartesian coordinates and a weight that depends on the van der Waals radius of the atom, determined using the CHARMM36m force field. A contact occurs whenever two atoms share a common face in the tessellation. The interface between molecular groups is quantified by their polygonal surface area. The type of contacts in the interface determines the nature of the interface: hydrophobic contacts correspond to contacts between carbon atoms exclusively, while electrostatic contacts involve N, O, S, and P atoms; hydrogen bonds and salt bridges both belong to this category. For each aa of interest, we calculated the frequency of contacts with neighboring residues and lipids as determined by the VLDM program. The occurrences of these contacts were then plotted using in‐house R scripts.

### 2.3 3D Structure Visualization and Sequence Alignment Rendering

3D structures were manipulated using Chimera 1.13.1 [39] and VMD [40]. Sequence alignment was rendered using ESPript [41].

### 2.4 Genetic Analysis

Sanger sequencing was used to analyze the coding exons and intron/exon junctions of the *SLC40A1* gene, as well as those of the *HFE*, *HFE2*, *HAMP*, and *TFR2* genes, which are associated with rare forms of hemochromatosis; details available on request. The proband was negative for the p.Cys282Tyr causal mutation in *HFE*.

Informed consent for molecular studies and clinical data publication was obtained from the proband, in accordance with the Declaration of Helsinki; in line with French ethical guidelines, the Clinical Research Ethics Committee of the University Hospital of Brest approved the study.

### 2.5 Plasmid Constructs

All HsFPN1 variations were introduced by the QuikChange Lightning Site‐Directed Mutagenesis Kit according to the manufacturer's instructions (Agilent Technologies Cat# 210518) in the pIRES_HsFPN1‐V5_CD8 and pcDNA3.1_HsFPN1‐V5 plasmid constructions, previously described [14]. The full‐length *SLC40A1* cDNA was checked by sequencing analyses after each site‐directed mutagenesis.

### 2.6 Culture and Transfection of Human Epithelial Kidney (HEK)293 T Cells

HEK293T cells, from the American Type Culture Collection (ATCC Cat# CRL‐11268, RRID:CVCL_1926), were incubated at 37°C in a 5% CO_2_ humidified atmosphere and propagated in Dulbecco's modified Eagle's medium (DMEM; Lonza Cat# 12‐604) supplemented with 10% fetal bovine serum (Eurobio Cat# CVFSVF06 01). Cells were tested for mycoplasma contamination every 3 months using the MycoAlert PLUS Mycoplasma Detection Kit (Lonza Cat# LT07‐703). HEK293T cells were transiently transfected using JetPEI (Polyplus Cat# 101‐40N), according to the manufacturer's instructions, and a 2:1 transfection reagent (μL)/plasmid DNA ratio (μg).

### 2.7 Isolation of Cell Surface Proteins and Western Blot Analysis

Membrane proteins were biotinylated and purified using the Pierce Cell Surface Protein Isolation Kit according to the manufacturer's instructions (Thermo Fisher Scientific Cat# 89881). Western blot analysis was performed using a mouse monoclonal primary antibody against V5 (Thermo Fisher Scientific Cat# R960‐25, RRID:AB_2556564) and then a polyclonal goat anti‐mouse immunoglobulins/HRP (Agilent Cat# P0447, RRID:AB_2617137) as the secondary antibody. The membranes were incubated with the Immobilon Forte Western HRP substrate as directed by the manufacturer (Merck Millipore Cat# WBLUF0100) and digitized for pattern analysis using the GeneGnome XRQ (Syngene GGNOME‐XRQ‐NPC).

### 2.8 Densitometry Study

To quantitate ferroportin 1 expression levels on the cell surface, the optical density of HsFPN1/V5 and HLA‐A/V5 protein bands was measured from the digital images by the GeneTools software (Syngene). The expression of ferroportin 1 was then normalized with cotransfected HLA(A).

### 2.9 Flow Cytometry Experiments

Flow cytometry experiments were led as previously described [14]. Cells were analyzed using a Navios flow cytometer (Beckman Coulter) and the Flowlogic software (Miltenyi Biotec). Events were gated to exclude cell debris and aggregates, and to select cells with the desired levels of CD8 and HsFPN1‐V5 expression; the windows were set up to exclude CD8‐ or HsFPN1‐V5‐negative cells (using mock‐transfected cells as control), as well as cells with very high fluorescent intensity.

### 2.10 ^55^Fe Measurements

Intracellular ^55^Fe measurements—^55^Fe loading of human apotransferrin was performed as previously described [42]. HEK293T cells (1.7 × 10^5^ cells per well in 12‐well plates) were transfected with wild‐type or mutated pcDNA3.1_HsFPN1‐V5 constructs for 24 h, before being cultured in Pro293a‐CDM serum‐free medium (Lonza Cat# BEBP12‐764Q) and preloaded with 20 μg/mL ^55^Fe‐transferrin for 16 h. Each pcDNA3.1_FPN‐V5 construct was co‐delivered with the pSV‐β‐Galactosidase control vector (Promega Cat# E1081). After being washed, the cells were harvested with trypsin, mixed with liquid scintillation fluid (Ultima Gold MV, Perkin Elmer Cat# 6013329) and counted for 2 min in a TRI‐CARB 1600 CA scintillation counter (Perkin Elmer). ^55^Fe radioactivity was normalized on total protein content and β‐GAL activity. The 5 independent experiments were performed in triplicate. Each experiment was normalized by the control “No HsFPN1”. For this purpose, all values (including control) were divided by the mean of the control to preserve relative variability in the control cohort.

^55^Fe release measurements—Transfected HEK293T cells cultured in Pro293a‐CDM serum‐free medium and preloaded with 20 μg/mL ^55^Fe‐transferrin for 16 h (see above) were washed with PBS and cultured in Pro293a‐CDM serum‐free medium for 36 h. ^55^Fe exported into the supernatant was then collected, mixed with liquid scintillation fluid, and counted for 2 min in a TRI‐CARB 1600 CA scintillation counter. Percentage ^55^Fe export was calculated using the following formula: (^55^Fe in the supernatant at 36 h, divided by cellular ^55^Fe at time zero) × 100. The 5 independent experiments were performed in triplicate.

### 2.11 Statistical Analyses

Data are shown as mean ± standard deviation (SD) and representative of at least 3 independent experiments. Each dataset was verified for Gaussian distribution using the Shapiro–Wilk test (*ɑ* = 0.05). Statistical comparisons between groups were analyzed for significance by unpaired two‐tailed Student's *t*‐test or one‐way ANOVA with Dunnett's post hoc test. A *p* value < 0.05 was considered statistically significant. Statistical analyses were conducted using GraphPad Prism 9.5.0 (RRID:SCR_002798) (GraphPad Software). *p*‐values are indicated in the figures with *p* < 0.05 (*), < 0.01 (**), < 0.001 (***) and < 0.0001 (****).

## 3 Results

### 3.1 Molecular Dynamics (MD) Simulations

We have performed MD simulations using the now available experimental 3D structure of HsFPN1 in an apo, outward‐facing state (PDB ID 6W4S, full‐length protein lacking amino acids (aa) 1–16 (N‐terminus), 239–290 (inter‐lobe hinge), aa 394–450 (TM8‐TM9 loop)) [16]. The protein was embedded in a bilayer of POPC (1‐palmitoyl‐2‐oleoyl‐sn‐glycero‐3‐phosphocholine), POPE (1‐palmitoyl‐2‐oleoyl‐sn‐glycero‐3‐phosphoethanolamine), POPS (1‐palmitoyl‐2‐oleoyl‐sn‐glycero‐3‐phospho‐L‐serine) and cholesterol (CHOL) in a 2:1:1:1 ratio (4× ~1000 ns of MD simulation), designed to reflect a complex, physiologically relevant membrane environment. Root Mean Square Deviation (RMSD) values for the C‐alpha atoms were calculated to assess the overall stability of the system after equilibration.

High and variable RMSD values were initially observed for the entire sequence (Figure S2‐IA). To investigate the source of this variability, Root Mean Square Fluctuation (RMSF) analysis was conducted to quantify amino acid displacements during the MD simulations, revealing highly flexible loops (Figure S2‐IB). Excluding these flexible regions from the RMSD calculation significantly reduced the variability, yielding lower and more stable RMSD values (Figure S2‐IC). Importantly, no large conformational changes were detected in the protein core, with the OF conformation maintained across all MD simulation replicas. Additionally, the characteristics of the membrane remained stable throughout the simulations (Figure S3A).

Our analysis focused on the two networks of charged and polar amino acids, referred to as networks 1 and 2 in Figures 1 and S1.

#### 3.1.1 A Stable Network 1 Involving the Arg88, Asp157 and Arg489 Trio

**FIGURE 2:**
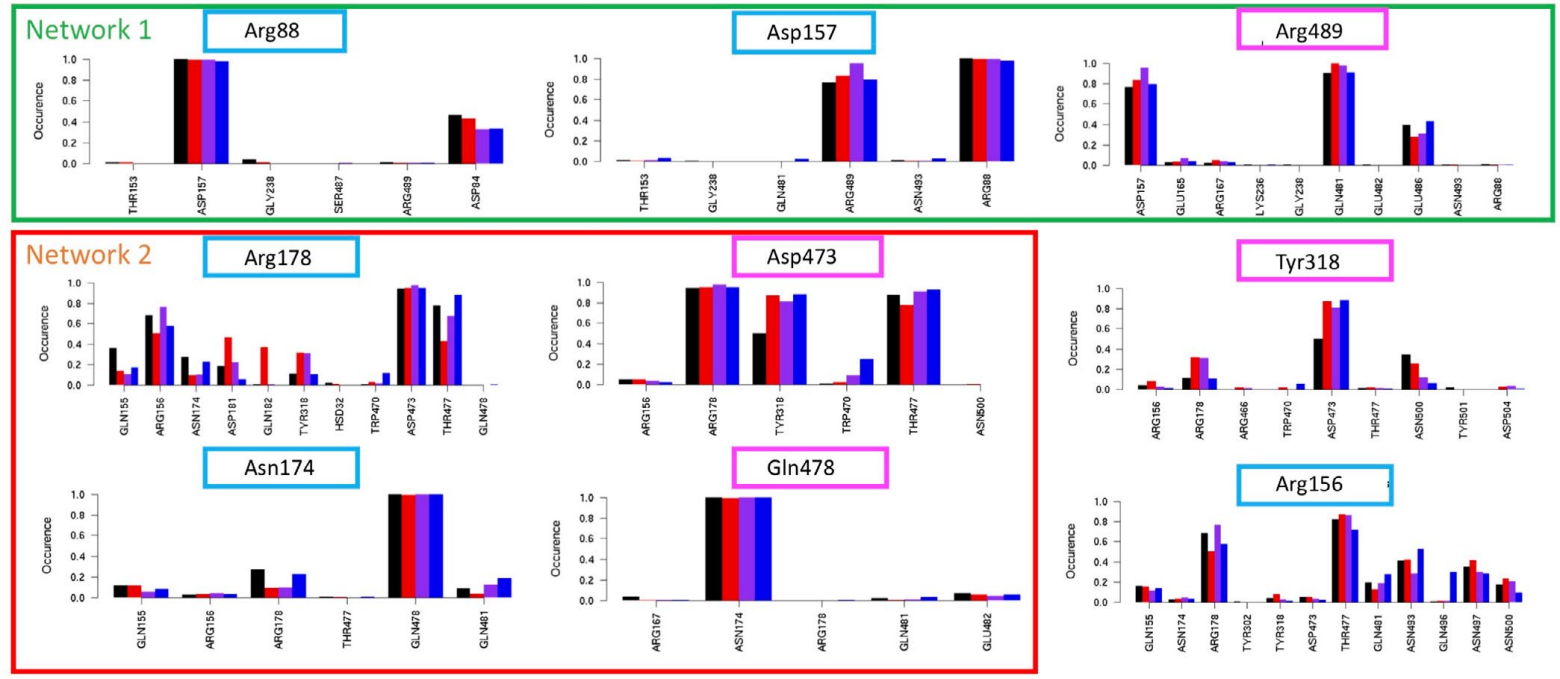
Occurrences (percentages) of electrostatic contacts observed along the MD simulations for the amino acids participating in the networks of the HsFPN1 intracellular gate. The four colors are related to the four MD simulation replica.

**FIGURE 3:**
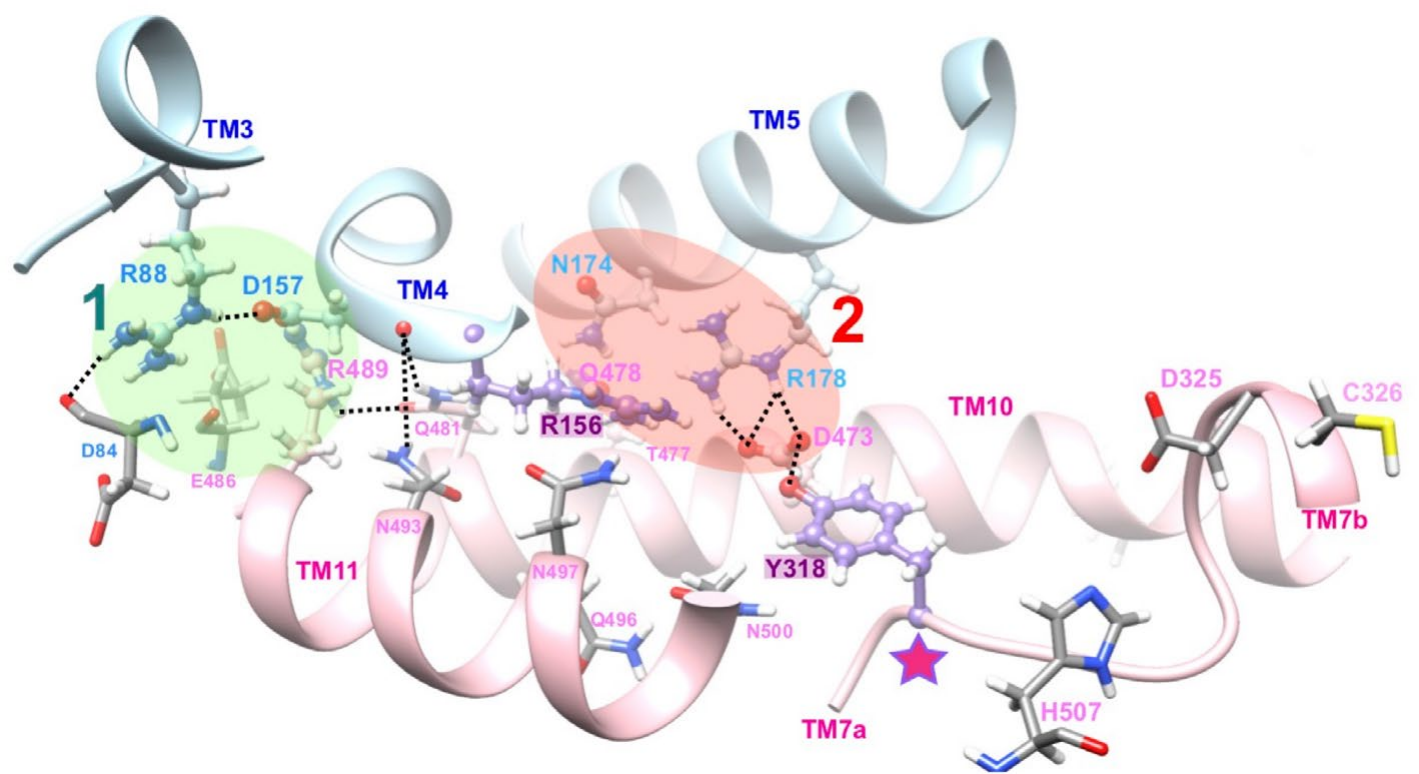
Networks of non‐covalent bonds in the intracellular gate of HsFPN1: Focus on Tyr318 and Arg156. Positions of Tyr(Y)318 and Arg(R)156 (purple) within the intracellular gate network depicted in Figure 1, after 1000 ns of MD simulation (replica 3). Coloring is the same as in Figure 1. The unwound segment of TM7 is highlighted with a star, with amino acids participating in the C‐lobe metal binding site depicted in atomic details (Asp (D) 325 and Cys (C) 326 on TM7b, His(H)507 on TM11). Amino acid names are indicated using the one‐letter code.

The salt bridges observed in the experimental 3D structure, which link the three amino acids of network 1 (Arg88, Asp157 and Arg489, green circle on Figure 1), were maintained along the MD simulations. Indeed, Arg88 on TM3 establishes stable salt bridges with Asp157 on TM4 (Figures 2 and 3). Albeit less frequent, contacts are also observed between Arg88 and Asp84 (TM2), another well‐conserved amino acid of the A‐motif. Asp157 also stably contacts Arg489 (TM11), which in turn appears to establish additional and frequent contacts with Gln481 (TM10), and to a lesser extent, with Glu486 (TM11), two amino acids between which it is sandwiched. These observations are consistent with our previous studies while adding further details to the dynamics of network 1, involving transient interactions of the previously identified Arg88‐Asp157‐Arg489 stable network with Asp84, Gln481, and Glu486.

#### 3.1.2 Arg156 and Tyr318, Located in the Vicinity of the Network 2 Arg178‐Asp473 Salt‐Bridge, are Novel Critical Actors of the HsFPN1 Iron Export Function

In network 2 (red circle on Figure 1), Arg178 (TM5) forms salt‐bridges with Asp473 (TM10), which are stable throughout the MD simulations (Figures 2 and 3). The Arg178‐Asp473 pair forms the wall of the deep cavity displayed in the OF conformation and is close to other polar amino acids, with Arg156 (TM4) and Tyr318 (TM7) being the most prominent. More precisely, the hydroxyl side chain of Tyr318 establishes frequent H bonds with the oxygen atom (O1 or O2) of Asp473, a feature which was not observed in the cryo‐EM 3D structure. Tyr318 is located at the end of TM7a, at the beginning of the unwound region linking TM7a to the shorter TM7b (Figure 3). The side chain of Arg156, one amino acid before Asp157 in network 1, is stably oriented, with its guanidium group pointing towards the Arg178‐Asp473 salt‐bridge. In particular, its side chain appears to be surrounded by a sheath made of the side chains of, on the one hand, Asn493, Gln496, Asn497, and Asn500 on TM11 and, on the other hand, Thr477 on TM10 (Figure 3). Thus, the MD simulations performed here suggested novel actors of the HsFPN1 inner gate (shaded purple in Figure 3), which not only connect the two networks (Arg156) but also establish a direct connection to the unwound region of TM7 (Tyr318), which is involved in iron binding in the C lobe.

#### 3.1.3 The Importance of Gln478 as a Stable Partner of Asn174 in Network 2

In the same network 2, Asn174 on TM5 formed H bonds with Gln481 in the experimental 3D structure. However, the MD simulations led to a rapid shift in the interaction network, as Asn174 formed very stable H bonds with Gln478 on TM10 (Figures 2 and 3). This interaction was observed in our earlier MD simulation of a model of the HsFPN1 3D structure built using the crystal structure of the bacterial homolog BbFpn1 [14]. The critical role of Gln478 in the establishment of network 2 was further supported by a directed mutagenesis approach, in which it was replaced by an alanine (a non‐polar amino acid, with high propensity for alpha‐helices), and subsequent in vitro assays [14]. In contrast to the Arg178‐Asp473 pair, the Asn174‐Gln478 pair does not form significant electrostatic contacts with other amino acids along the MD simulations (Figure 2).

#### 3.1.4 Irregularities in TM Helices Add to the Asymmetry of the Inner Gate's Bond Networks

**FIGURE 4:**
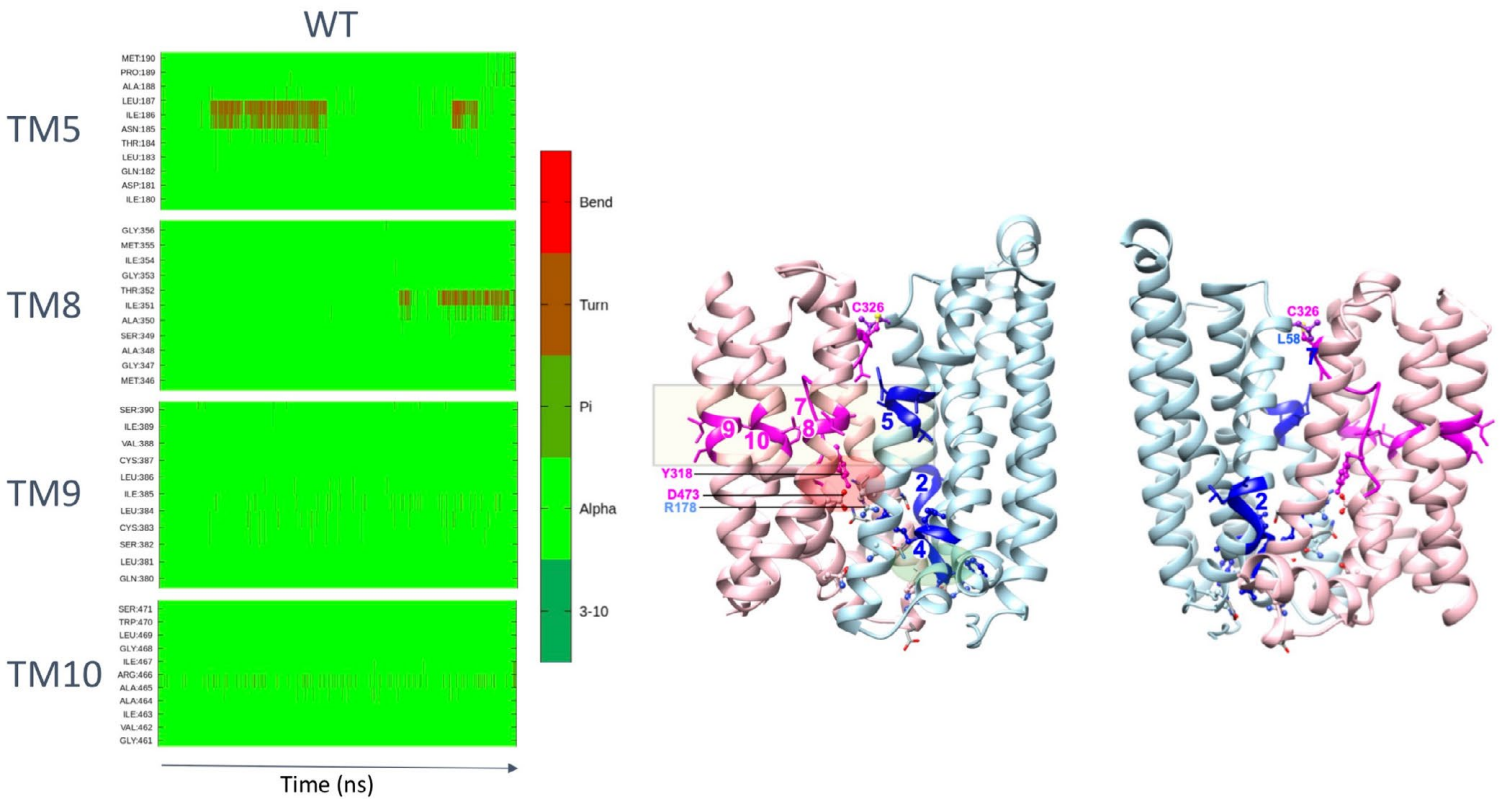
Alpha helix irregularities along the MD simulations. Hydrogen bond patterns were followed along the MD simulations (here WT HsFPN1, replica 3), in order to highlight local irregularities (dark green, brown and red) relative to the alpha‐helix pattern (green). A cluster of amino acids (magenta/blue—boxed) identified on helices TM5, TM8, TM9 and TM10 is shown on the 3D structure of WT HsFPN1 after 1000 ns MD simulation (two orthogonal views), as well as regions highlighted on TM4, TM7 and TM2 (blue). Amino acid names are indicated on the 3D structure using the one‐letter code.

In order to further understand the internal dynamics of the protein, we analyzed the MD simulations by following the hydrogen bond networks within the TM helices. Helix irregularities are known to confer plasticity in the structure and facilitate local rearrangements required to move between different conformational states [43, 44]. Some local irregularities relative to the alpha‐helix pattern (i.e., 3_10_ helix or even turn or bend) were observed in some HsFPN1 TM helices (Figure 4, illustrated for replica 3—with amino acids in alpha helix conformation in green in the plot at left). Most of them, involving amino acids from TM5 (aa 184–187), TM8 (aa 350–352), TM9 (aa 382–385) and TM10 (aa 465–466), are clustered in a region (Figure 4, box including the segments colored dark blue (lobe N) and magenta (lobe C) in the 3D structure shown at right) in the vicinity of the unwinding in TM7 (aa 316–326, magenta), next to Tyr318 and the inner gate network 2 Arg178–Asp473 salt‐bridge. Thus, the asymmetry observed at the level of the two inner gate's bond networks extends into a connected and dynamic TM slice. Of note is the presence of another distant local irregularity in TM2 (aa 74–75, dark blue), where bending occurs leading to a concerted movement of the upper part of TM2 with the upper part of TM7 (contact between TM2 Leu58 and TM7 Cys326) (Figure 4). Finally, another irregularity also occurs in TM4, at the level of the Arg156–Asp157 pair, where two networks of non‐covalent bonds are connected (Figure 4).

### 3.2 Functional Characterization of the p.Arg156Ala and p.Tyr318Ala Mutants

We aimed to verify the importance of residues Arg156 and Tyr318 by changing them to alanine and assessing the effect of each variation on HsFPN1 activity. The loss‐of‐function mutation p.Val162del, reported to reduce cell surface localization (and thus iron efflux) [45], and the p.Gln478Ala mutation, which we previously demonstrated to impair the iron‐exporting ability of HsFPN1 without affecting its plasma membrane expression [14], were used as negative controls. We used HEK293T cells as a model. These cells were used in the seminal study of the role of FPN1 in iron metabolism and its regulation by hepcidin [3] and, since then, have been adopted by a majority of researchers in the field. There may be differences in the evaluation of FPN1 mutants across various cell types, particularly between HEK293T and macrophage‐derived cells compared to enterocyte‐derived cells [46]. However, this remains a hypothesis that has not been thoroughly investigated.

**FIGURE 5:**
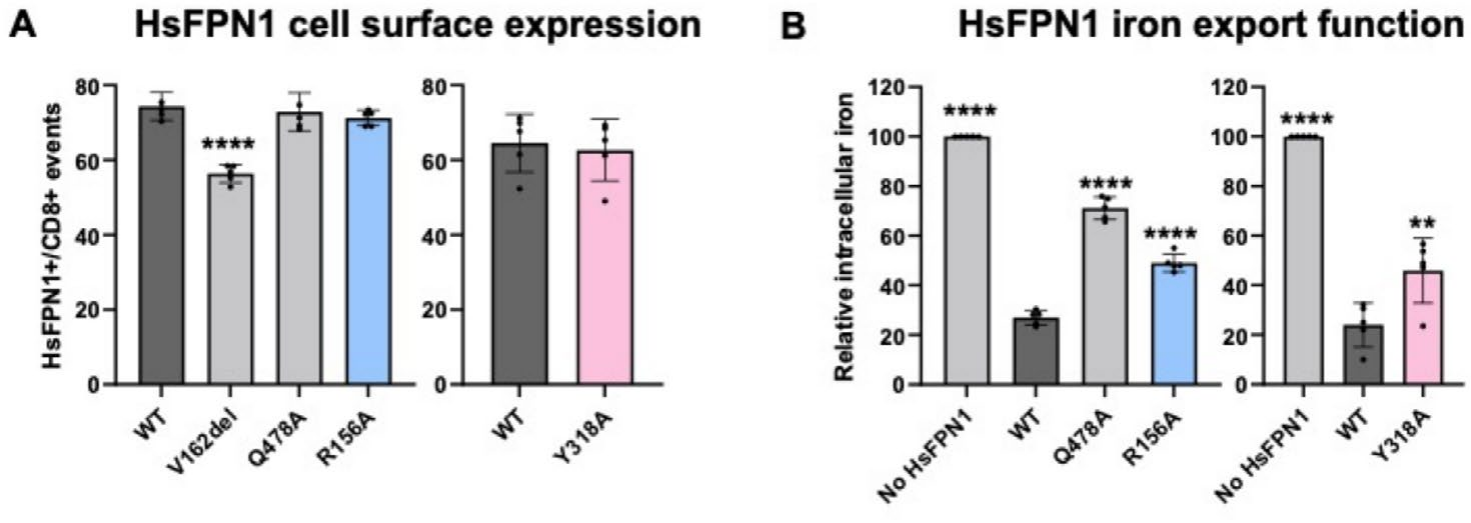
Effect on cell‐surface expression and iron export of alanine substitutions for amino acids predicted to be involved in interactions between the N‐ and the C‐lobe of HsFPN1 in the outward‐facing state. (A) HEK293T cells were transiently transfected with the bicistronic pIRES2 plasmid encoding both full‐length HsFPN1 and the cluster of differentiation antigen 8 (CD8). A 14‐amino‐acid V5 epitope (Gly‐Lys‐Pro‐Ile‐Pro‐Asn‐Pro‐Leu‐Leu‐Gly‐Leu‐Asp‐Ser‐Thr) was inserted in the 5th extracellular loop (ES5) of HsFPN1 to allow plasma membrane detection. After 36 h, cells were double‐stained for CD8 (APC) and the HsFPN1‐V5 fusion protein (FITC) and analyzed by 2‐color flow cytometry. Data are presented as percentage of HsFPN1‐positive over CD8‐positive events. Each bar represents the mean ± standard deviation of 5 independent experiments. One‐way ANOVA followed by Dunnett's multiple comparisons test (control group = WT): *p* < 0.0001 (****) (left panel), unpaired two‐tailed Student's *t*‐test (right panel). (B) HEK293T cells were transfected with pcDNA3.1‐HsFPN1‐V5‐His vectors, grown for 24 h, and then fed with 20 μg/mL ^55^Fe‐transferrin for 16 h. Cells were then washed, and counted. Counts per minute (cpm) were normalized by total protein and β–Gal activity. Each bar represents the mean ± standard deviation of 5 independent experiments. One‐way ANOVA followed by Dunnett's multiple comparisons test (control group = WT): *p* < 0.01 (**) and < 0.0001 (****).

The cell surface expression of the p.Arg156Ala and p.Tyr318Ala mutants was evaluated in transiently transfected HEK293T cells by flow cytometry, as previously reported [14]. The co‐overexpressed cluster of differentiation 8 (CD8), which is not normally found in HEK293T cells, enabled correction for differences in transfection efficiency between samples. As expected, important differences were observed in the proportions of HsFPN1‐WT+/CD8+ and HsFPN1‐Val162del+/CD8+ cells (*p* < 0.0001), confirming the p.Val162del mutant mislocalization (Figure 5A). In contrast, the p.Arg156Ala and p.Tyr318Ala mutants reached the plasma membrane correctly.

The iron‐exporting function of the p.Arg156Ala and p.Tyr318Ala mutants was assessed by measuring the accumulation of radioactively labeled iron in HEK293T cells co‐transfected with pcDNA3.1‐HsFPN1‐V5 plasmids and a normalization vector encoding the ß‐gal enzyme, as also previously reported [14]. This method was preferred to iron export measurements to better appreciate the effects of the different variations, particularly those that lead to modest effects; Figure S4 shows that this method gives results comparable to those obtained with direct measurement of iron export (differences observed between the wild‐type protein and the p.Gln478Ala control), but with significantly lower experimental variability. Cells transfected with the commercial pcDNA3.1‐V5‐His empty vector served as a negative control (No HsFPN1). As shown in Figure 5B, cells overexpressing the WT HsFPN1 protein displayed 3‐ to 4‐fold lower iron accumulation. None of the p.Gln478Ala, p.Arg156Ala, and p.Tyr318Ala mutants were able to export iron in amounts comparable with WT HsFPN1.

These results provide compelling support for the implication of Arg156 and Tyr318 in the HsFPN1 iron export mechanism.

### 3.3 p.Gln478Arg as a New Disease‐Causing Mutation

While we were deepening our characterization of the HsFPN1 inner gate, a 45‐year‐old patient was referred to us because of unexplained hyperferritinemia. His phenotype was suggestive of ferroportin disease. Indeed, the patient presented with a serum ferritin concentration exceeding 1000 μg/L (1200) and a transferrin saturation of less than 45% when he was 43 years old. Normal blood pressure, BMI (175 cm, 65 kg), and lipid metabolism argue against a metabolic syndrome. Aspartate aminotransferase (52 UI/L), alanine aminotransferase (69 UI/L) and gamma‐glutamyl transferase (69 UI/l) were moderately increased, while hepatic elasticity measures were within the normal range. Magnetic Resonance Imaging revealed a reduction in the signal intensity of the liver, consistent with a significant hepatic iron overload (Hepatic Iron content estimated at 160 μmol/g). No information was available on the existence of a hyposignal in the spleen.

We identified a novel A>G transition at position 1433 of the S*LC40A1* coding region (NM_014585.5:c.1433 A>G), which changes glutamine 478 to arginine (p.Gln478Arg). This novel missense variation, which was detected at the heterozygous state (Figure S5), is not referenced in the following international databases: gnomAD (Genome Aggregation Database), ExAC (Exome Aggregation Consortium) and HGMD (Human Gene Mutation Database). Furthermore, it is absent in FREX (French Exome Project; http://lysine.univ‐brest.fr/FrExAC/), meaning that the frequency of the c.1433G allele in the French population is estimated at 0.0009 on Wald's method (not detected on the 1148 tested chromosomes).

**FIGURE 6:**
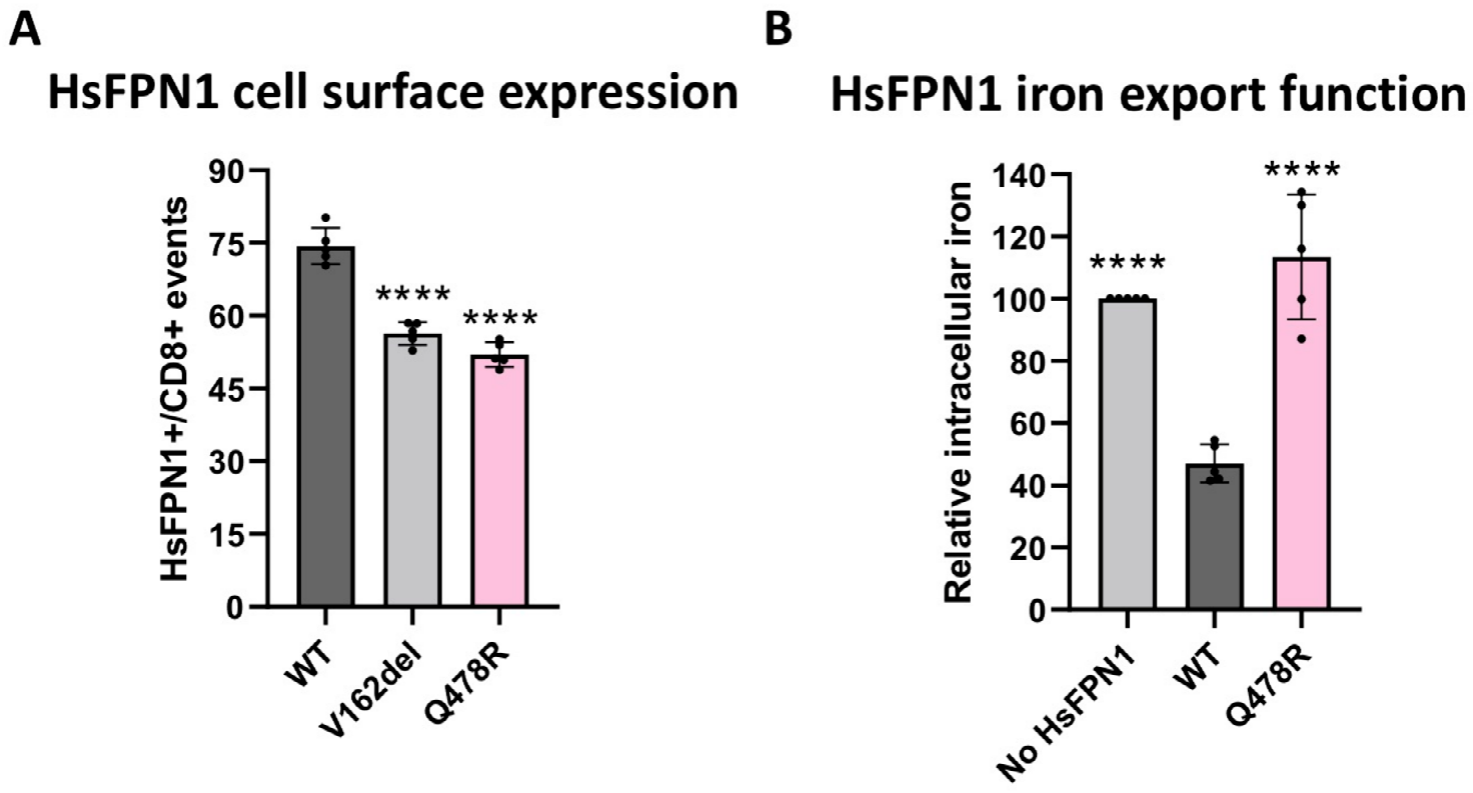
In vitro evaluation of the p.Gln478Arg variant. (A) HEK293T cells were transiently transfected with the bicistronic pIRES2 plasmid encoding both full‐length HsFPN1 and the cluster of differentiation antigen 8 (CD8). A 14‐amino‐acid V5 epitope was inserted in the 5th extracellular loop (ES5) of HsFPN1 to allow plasma membrane detection. After 36 h, cells were double‐stained for CD8 (APC) and the HsFPN1‐V5 fusion protein (FITC) and analyzed by 2‐color flow cytometry. Data are presented as percentage of HsFPN1‐positive over CD8‐positive events. Each bar represents the mean ± standard deviation of 5 independent experiments. One‐way ANOVA followed by Dunnett's multiple comparisons test (control group = WT): *p* < 0.0001 (****). (B) HEK293T cells were transfected with pcDNA3.1‐HsFPN1‐V5‐His vectors, grown for 24 h, and then fed with 20 μg/mL ^55^Fe‐transferrin for 16 h. Cells were then washed, and counted. Counts per minute (cpm) were normalized by total protein and β–Gal activity. Each bar represents the mean ± standard deviation of 5 independent experiments. One‐way ANOVA followed by Dunnett's multiple comparisons test (control group = WT): *p* < 0.0001 (****).

The functional significance of the p.Gln478Arg variant was investigated in vitro as presented above for the p.Arg156Ala, p.Tyr318Ala, and p.Gln478Ala mutants. It was found to be deeply depleted on the surface of HEK293T cells (Figure 6A). This was confirmed using another method (isolation of cell surface proteins and Western Blot analysis; Figure S6). As a result, its ability to export iron was considerably reduced (Figure 6B).

**FIGURE 7:**
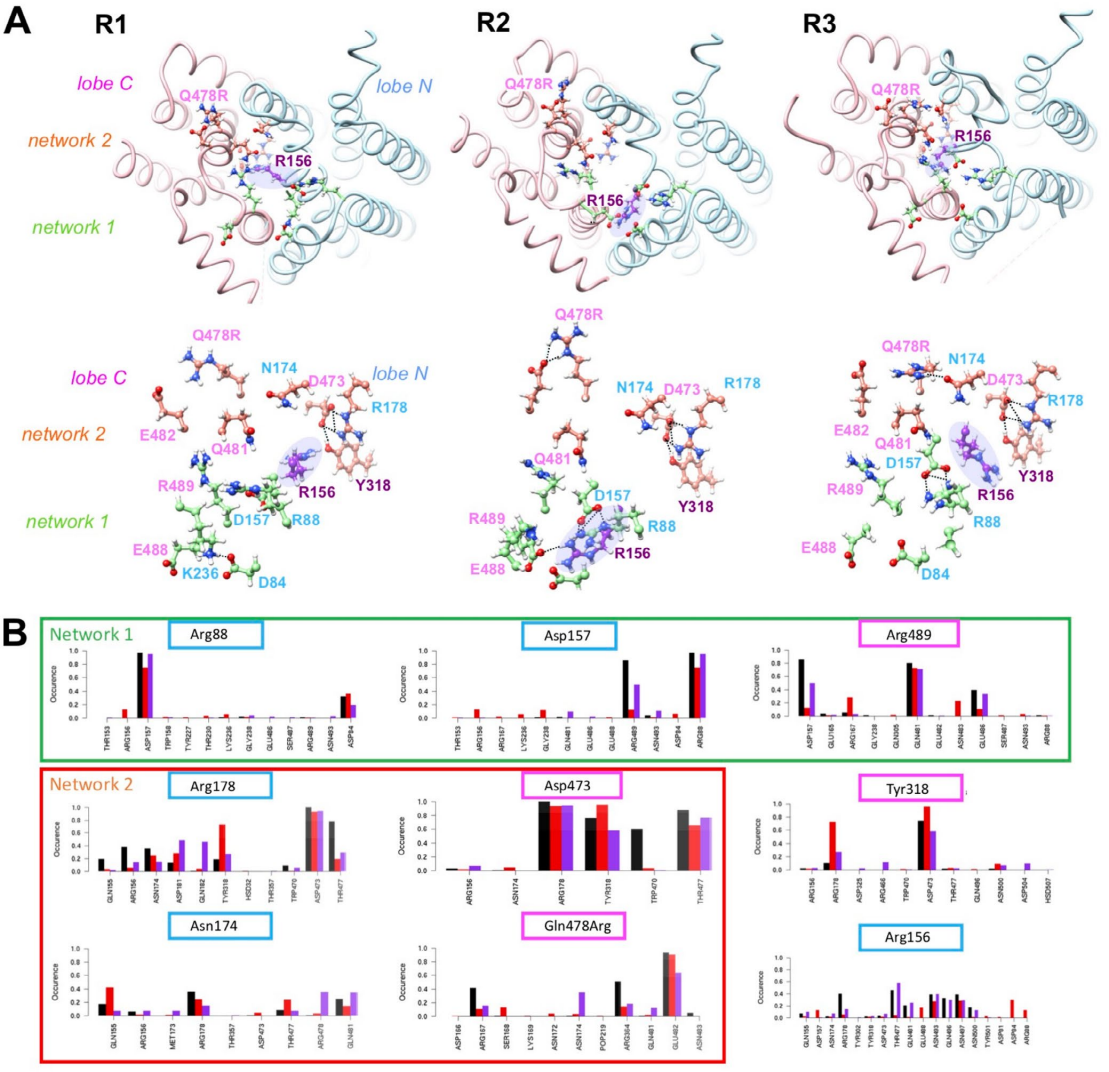
Impact of the p.Gln478Arg variation on the HsFPN1 3D structure. (A) View of amino acids of the second intracellular gate network (illustrated after 1000 ns MD simulation for the three replica R1 to R3), including the Gln(Q)478Arg(R) variation. A shift of R156 (shaded circle) towards network 1 is observed in replica 2. (B) Occurrences (percentages) of electrostatic contacts observed along the MD simulations for the amino acids of networks 1 and 2 (to be compared with Figure 2). The three colors are related to the three MD simulation replica. Amino acid names are indicated in panel A using the one‐letter code.

These observations were correlated with the results of 3 replicas of ~1000 ns MD simulations of the mutated protein, suggesting a strong impact on the whole system and more particularly on the inter‐lobe network (Figure 7). First, RMSD values (calculated with exclusion of loop and hinge regions) are globally higher in the mutated protein compared to the WT MD simulations, while RMSF analysis highlighted more mobile regions (Figure S2‐II). Additionally, slight differences in the membrane properties are also observed in two MD simulations of the mutated protein (Figure S3B). At the level of the inner gate network 2, the H‐bonds between Gln478 (TM10) and Asn174 (TM5) observed in the WT protein were largely abolished in the mutated protein, leaving Asn174 mainly disconnected from the inter‐lobe network (Figure 7). Moreover, the position of the Arg478 (TM10) side chain is constrained by the proximity of the TM5 helix, leading to an orientation towards the membrane‐cytosol interface and frequent stable bonds (salt‐bridges) with Glu482 on the same helix (Figure 7). Despite these changes, the Arg178‐Asp473 salt‐bridge and contacts with neighboring residues remained stable in the time frame of the MD simulations (Figure 7B). Nevertheless, at the end of one MD simulation, a significant change is observed in the position of Arg156, leading to interaction with network 1 residues (Glu488, Asp84, Asp157) and consequently to an uncoupling of the two networks (Figure 7). Finally, the irregularity occurring in TM4 at the level of Arg156‐Asp157 appears more pronounced in some parts of the MD simulations performed with the HsFPN1 p.Gln478Arg mutant (Figure S7). This lends further support to the hypothesis that this region is a critical actor in the conformational changes associated with the disruption of the inner gate.

Altogether, these data confirm the loss‐of‐function nature of the p.Gln478Arg missense variation. Its effect could be direct or indirect (through an effect on protein stability in the membrane) with possible perturbations to both network 2 and the connection to network 1.

## 4 Discussion

Ferroportin 1 is the only known non‐heme iron exporter in vertebrates. While adopting a canonical MFS fold, this multipass membrane protein has evolved to acquire particular characteristics. The most remarkable feature is its regulation by hepcidin, the systemic iron‐regulatory hormone [47]. This has important consequences in humans, since the HsFPN1‐hepcidin axis has been implicated in the pathogenesis of different forms of iron overload diseases and iron‐restricted anemia. Downregulation of HsFPN1 has also been detected in various types of cancers, where it is thought to promote iron retention and cell proliferation [5, 48]. Conversely, loss of HsFPN1 in cells can induce iron‐dependent cell death, known as ferroptosis [49, 50, 51].

**FIGURE 8:**
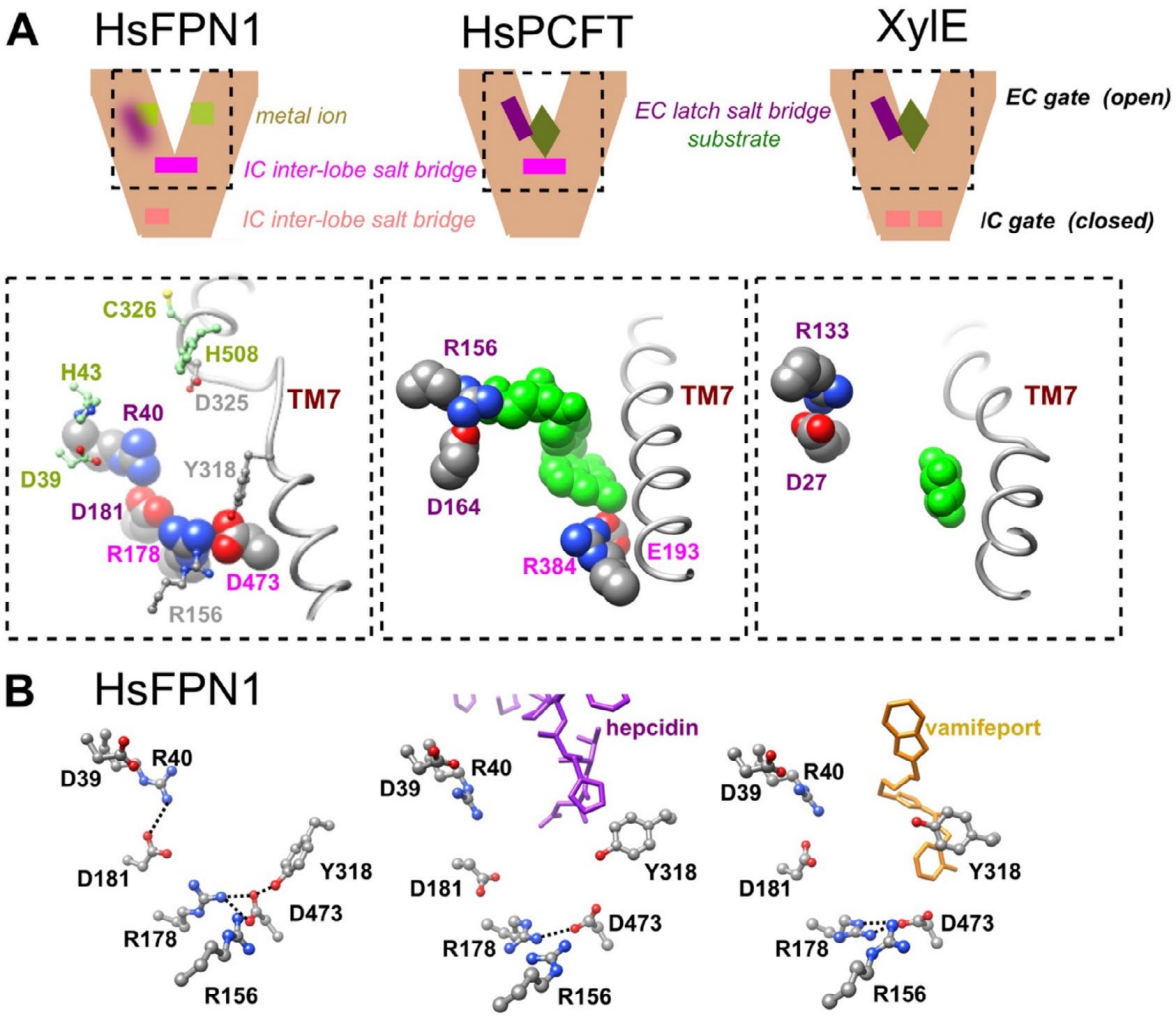
The outward‐facing cavity of HsFPN1. (A) Comparison of the HsFPN1 3D structure after MD simulation with the proton‐coupled MFS transporter HsPCFT bound to pemetrexed (green) (pdb 7bc7) [52] and the proton xylose transporter XylE bound to d‐xylose (green) (pdb 4GBY) [53]. (B) Comparison of the HsFPN1 3D structure after MD simulation with the 3D structure of HsFPN1 in complex with hepcidin (pdb 6WBV, [16]) and with vamifeport (pdb 8C03, [17]).

The assembly and stabilization of FPN1 in the plasma membrane in an OF conformation is a preliminary and essential step in the mechanism of ferrous iron export in humans and other vertebrates [15, 16, 18]. The OF conformation, which also enables the binding of hepcidin [16, 18], is highly dependent on non‐covalent interactions between specific residues at the cytoplasmic side of the lipid bilayer, connecting the N and C lobes and forming the inner gate. In MFS transporters, the inner gates are shaped by the close packing of TM4‐TM5 and TM10‐TM11, sealing a large polar, clamshell‐like cavity open to the extracellular side, with the substrate binding site at its center [8, 11]. HsFPN1 conforms to this mold, with an overall negative electrostatic potential of the cavity [18], while presenting distinctive features (Figure 8A). First, its cavity contains two conserved metal ion binding sites, which are disconnected and in which coordination is ensured by two residues (N‐lobe: Asp39 and His43 from TM1; C‐lobe: Cys326 from TM7b and His508 from TM11). This is different from iron binding sites identified in other transition metal iron transporters of known structures [16, 18]. Second, a discontinuity is observed in the transmembrane helix TM7 (overlapping the C‐lobe metal ion binding site). As with sugar MFS transporters (reviewed in [8]), this likely provides the structural adaptability needed for conformational changes [15, 16, 18, 54]. Third, the salt bridge that forms between Arg178 (TM5) and Asp473 (TM11) is not only distant from the membrane–cytosol interface, but also forms the floor of the deep cavity displayed in the OF conformation.

Our observations point out the Arg156 and Tyr318 residues as new important actors in the functional architecture of the HsFPN1 inner gate (Figures 2, 5 and 8A left panel). Indeed, Tyr318 connects the central Arg178‐Asp473 salt bridge to the C‐lobe metal ion‐binding site, at the level of the unwound TM7 segment, while Arg156 connects it to a second network of non‐covalent bonds positioned at the membrane‐cytosol interface (here defined as network 1; Figure 1). The two residues may provide an environment favorable to the formation of the Arg178‐Asp473 salt bridge and/or its disruption. For instance, the polarizable electronic state of the aromatic tyrosine could help reduce the cost of desolvating the two charged partners of the salt bridge, by analogy to what has been proposed in the central gating region of the *E. coli* outer membrane protein OmpA [55]. Structures detailing the interactions between HsFPN1 and vamifeport, a small molecule inhibitor of iron export currently in clinical development for hereditary disorders, were published a year ago [17]. In this context, it is worth highlighting that the side chain of Tyr318 is drastically displaced from the position it occupies in the apo state (H‐bonded to Asp473), as a result of contacts made with both vamifeport and hepcidin in an OF state (Figure 8B). As both molecules block HsFPN1 in an OF state, it is tempting to suggest that Tyr318 plays a key role in the conformational switch of the inner gate. Tyr318, as well as Arg156, are not conserved in BbFpn1 (Figure S1), suggesting that they are also key actors in the specific mechanism that regulates FPN1 function in mammals. Finally, it is also worth mentioning here that we very recently identified Arg179 (one amino acid after Arg178) as a charged residue interacting with glycerophospholipids. This interaction between a basic amino acid and lipid polar heads is likely to result in a local deformation of the lipid bilayer in the direct vicinity of the Arg178‐Asp473 salt bridge, which could facilitate discrete conformational changes [56]. The in vitro results presented in Figure 5 confirm the importance of Arg156 and Tyr318 for the conformational dynamics of the HsFPN1 inner gate; expression of the mutated proteins at the cell surface is not altered, but transport activity is strongly affected. We should emphasize that we have chosen an indirect method to assess the iron export activity of HsFPN1. This approach aims to minimize experimental variability and better reveal partial effects. We verified the absence of experimental bias after comparing the wild‐type protein with the p.Gln478Ala mutant in direct and indirect measurements of HsFPN1 activity (Figure S4).

The present study sheds further light on the asymmetry of the bond networks present in the HsFPN1 inner gate. Analysis of the MD trajectories indicates that local irregularities in some TMs, clustered in the vicinity of the central network linking TM5 and TM10 and of the unwound segment of TM7, also contribute to that asymmetry and may be critical actors for the conformational transitions. Of note is that results from MD simulations should be considered with some caution, as there are missing parts (loops and linker between the two lobes) that can affect the overall dynamics of the system. However, the irregularities observed here are localized in the central part of the membrane‐spanning segments, without the ends of the TMs on either side of these segments undergoing alterations in their hydrogen bond patterns. The role of lipids in the overall dynamics of HsFPN1, not analyzed here, must also be taken into account.

We have compared the 3D structures of HsFPN1 and other MFS members, searching for potential similarities at the level of the inner gate bond networks and of the substrate‐binding cavity specific to the OF conformation. As shown in Figure 8A middle panel, the human proton‐coupled folate transporter PCFT (HsPCFT) has an inter‐lobe salt‐bridge (between Glu193 on TM5 and Arg384 on TM10) at the level of the substrate‐binding cavity floor (thus similar to the Arg178‐Asp473 pair in HsFPN1), overhanging the inner gate. This salt‐bridge stabilizes HsPCFT in the OF conformation, while protonation of the acidic residue in the presence of folate results in the breakage of this interaction, triggering the inner gate to open and allowing conformational changes for transport [52]. The proton motive force hypothesis for HsFPN1 has begun to be explored by only a few studies [18, 57, 58], without yet experimental evidence of the amino acids involved. Whether HsFPN1 Asp473 could be a critical site for (extracellular) proton‐induced activation is worth exploring. Protonation at HsFPN1 Asp473 could be associated with a conformational switch towards the IF state due to the loss of the inner gate stabilizing Asp473‐Arg178 salt bridge, thereby permitting cytosolic ferrous iron to access its binding sites. Continuing with HsPCFT, it is worth mentioning that an additional salt bridge is located higher up in the cavity (between Asp164 and Arg156) (Figure 8A). Upon folate binding (and protonation), this salt bridge breaks to promote closure of the extracellular gate [52]. This situation is similar to that reported for various proton‐coupled sugar transporters, as exemplified by XylE in which a salt bridge between Asp27 and Arg133 is described as participating in a latch mechanism [8, 59] (Figure 8A right panel). Such a mechanism could also be considered for HsFPN1, in which a salt bridge is also present in a comparable region, at the level of the N‐lobe metal binding site between Arg40 (TM1) and Asp181 (TM5) (Figure 8A,B). In this case, protonation of Arg40 might enable TM1 to converge on TM7b and close the extracellular gate. In this respect, we had previously proposed the possibility of an interaction between Arg40 (TM1, N‐lobe) and Asp325 (TM7b, C‐lobe) as a stabilizer of the inward facing conformation [19]. All these considerations on the involvement of protons in the HsFPN1 cycle remain speculative and need to be confirmed by experimental data.

We recently documented the spectra of disease‐causing mutations in the *SLC40A1* gene, confirming the association of 28 loss‐of‐function missense variations with ferroportin disease [60]. We argued that these pathogenic single amino acid changes are valuable indicators of HsFPN1 properties with important structural and functional consequences. Given the new observations made in the present study, we would like to insist on the fact that 13 of the 28 missense variations affect 7 critical residues of the inner gate in the transmembrane helices TM2, TM3, TM4, TM5, TM10, or TM11 (Asp84, Arg88, Asp157, Asn174, Arg178, Gln478, Arg489), and that 9 others affect 7 residues located in the direct vicinity of the two networks of non‐covalent bonds (Ala77, Gly80, Ile152, Trp158, Gly468, Gly490, Gly494) (Table 1). Asp181 is further regarded as a potential critical component for the formation of the HsFPN1 extracellular gate. Ferroportin disease is an autosomal‐dominant iron overload disorder characterized by cellular iron retention, principally in reticuloendothelial cells. This results in increased synthesis of the storage protein, ferritin, and relative iron deficiency (at least in the early stage of the disease). Diagnosis can occur early in life, but is most often made after age 40 [46, 60, 61, 62, 63]. Information about a French patient harboring the newly identified p.Gln478Arg substitution matches this phenotypic description very well. The results of the in vitro experiments shown in Figures 6 and S6 further confirm that p.Gln478Arg is a new loss‐of‐function missense variation, thus adding to the 23 disease‐causing mutations that are thought to destabilize the HsFPN1 inner gate and/or prevent conformational changes required for the outward‐ to inward‐facing transition.

**TABLE 1:** List of variations reported in patients with ferroportin disease that affect intracellular or extracellular gate residues.

Nucleotide change	Amino acid change	Localization in protein	References, pubmed identifier number (PMID)
c.230C>A	p.Ala77Asp	TM2	10471458, 15692071, 15831700, 15935710, 15951560, 16885049, 18160317, 19846751, 21396368, 23784628, 23943237, 24714983, 24859227, 30002125, 32450003
c.238G>A	p.Gly80Ser	TM2	16135412, 16885049, 17052926, 18420432, 21094556, 24370385, 24714983
c.239G>T	p.Gly80Val	TM2	16351644
c.252C>G	p.Asp84Glu	TM2	28681497
c.262A>G	p.Arg88Gly	TM3	17951290, 23943237, 24714 983
c.263G>C	p.Arg88Thr	TM3	16257244
c.263G>T	p.Arg88Ile	TM3	33385755
c.454A>T	p.Ile152Phe	TM4	18713659, 23784628
c.469G>T	p.Asp157Tyr	TM4	24714983
c.470A>G	p.Asp157Gly	TM4	12730114, 15935710, 15956209, 16457665, 24714983
c.469G>A	p.Asp157Asn	TM4	18177470, 29154924
c.470A>C	p.Asp157Ala	TM4	20230395, 20533066, 26183747
c.474G>T	p.Trp158Cys	TM4	21396368, 28110135
c.521A>T	p.Asn174Ile	TM5	16135412, 16885049, 24767627, 23784628, 29154924
c.533G>A	p.Arg178Gln	TM5	17951290, 17997113, 30002125, 32450003
c.541G>A	p.Asp181Asn	TM5	29154924
c.542A>T	p.Asp181Val	TM5	16351644, 24714983, 24767627, 24859227
c.1402G>A	p.Gly468Ser	TM10	18160816, 33341511
c.1433A>G	p.Gln478Arg	TM10	This study
c.1467A>C	p.Arg489Ser	TM11	16258219
c.1466G>A	p.Arg489Lys	TM11	19937651
c.1468G>A	p.Gly490Ser	TM11	17951290, 24714983
c.1469G>A	p.Gly490Asp	TM11	12873829, 15692071, 15956209, 24714983
c.1481G>A	p.Gly494Asp	TM11	34828384

In summary, this study extends our understanding of the conformational dynamics of the HsFPN1 inner gate. It sheds further light on the overall asymmetry of the inner gate bond network and to a not strictly conserved (Figure S1) charged/polar amino acids network linking TM5 and TM10. This one, located deeper in the lipid bilayer, may play a critical role in driving conformational transitions between the two extremes OF and IF conformations. This study also confirms that a substantial number of loss‐of‐function variations cluster in the HsFPN1 inner gate, modifying amino acids that are important for stability and transport activity. All these variations may explain ferroportin disease.

## Author Contributions

Marlene Le Tertre, Conceptualization, Formal analysis, Validation, Methodology, Writing – review and editing; Ahmad Elbahnsi, Conceptualization, Formal analysis, Funding acquisition, Validation, Methodology, Writing – review and editing; Cecile Ged, Clinical Investigation, Writing – review and editing; Kevin Uguen, Formal analysis, Writing – review and editing. Isabelle Gourlaouen, Methodology, Writing – review and editing; Claude Ferec, Funding acquisition, Writing – review and editing; Chandran Ka, Conceptualization, Validation, Methodology, Writing – review and editing; Gerald Le Gac, Conceptualization, Supervision, Validation, Funding acquisition, Writing – original draft; Isabelle Callebaut, Conceptualization, Supervision, Formal analysis, Funding acquisition, Writing – original draft.

## Conflicts of Interest

The authors declare no conflicts of interest.

## Supporting information


**Figure S1.** The inter‐lobe networks of the HsFPN1 intracellular gate: from bacteria to human.

**Figure S2.** Stability of the systems along MD simulations.

**Figure S3.** Membrane analysis.

**Figure S4.** Measurement of intracellular 55Fe retention versus 55Fe release in HEK293T cells not overexpressing or overexpressing wild‐type or p.Gln478Ala HsFPN1.

**Figure S5.** Sequencing electropherogram showing the SLC40A1 NM_014585.5 c.1433A>G nucleotide change.

**Figure S6.** Effect of the p.Gln478Arg variation on cell surface expression.

**Figure S7.** Alpha helix irregularities in TM4 along the MD simulations of WT and Q478R HsFPN1.

## Data Availability

The data that support the findings of this study are available on request from the corresponding author.
