# Supplementary material for: Identification of New Key Players for Ferrous Iron Export in the Asymmetric Inner Gate of Human Ferroportin 1
Source: FASEB J. 2025 Jul 10;39(14):e70821. doi: 10.1096/fj.202500790RR (PMC12246770; doi:10.1096/fj.202500790RR)
Supplement: Supplementary file 1 — Figure S1. The inter‐lobe networks of the HsFPN1 intracellular gate: from bacteria to human. [file FSB2-39-e70821-s004.pdf]

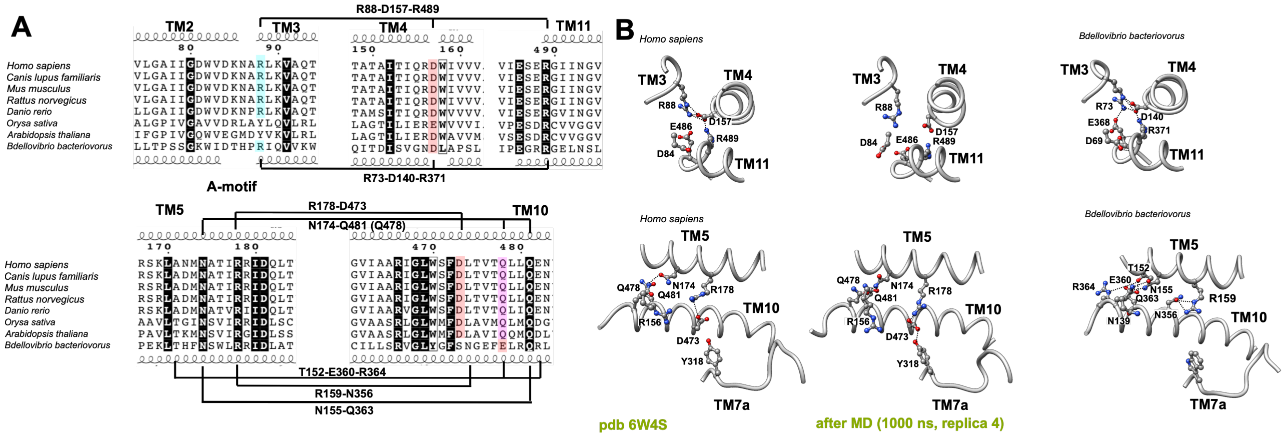

Supplementary Figure 1: The inter-lobe networks of the HsFPN1 intracellular gate: from bacteria to human. A. Alignment of ferroportin sequences from different species at the level of the first network (top) and second network (bottom), highlighting non-covalent bonds observed in the human FPN1 3D structure (top: PDB ID 6W4S, before and after MD simulation (1000 ns – replica 4, this study)) and of the *Bdellovibrio bacteriovorus* BbFpn 3D structure (bottom: PDB ID 5AYM). UniProt entries: Q9NP59 (*Homo sapiens*), E2RFJ3 (*Canis lupus familiaris*), Q9JHI9 (*Mus musculus*), Q923U9 (*Rattus norvegicus*), Q9I9R3 (*Danio rerio*), Q5Z922 (*Orysa sativa*), Q80905 (*Arabidopsis thaliana*), Q6MLJ0 (*Bdellovibrio bacteriovorus*). **B.** Illustration of these non-covalent bonds on the corresponding 3D structures.
