# Supplementary material for: Identification of New Key Players for Ferrous Iron Export in the Asymmetric Inner Gate of Human Ferroportin 1
Source: FASEB J. 2025 Jul 10;39(14):e70821. doi: 10.1096/fj.202500790RR (PMC12246770; doi:10.1096/fj.202500790RR)
Supplement: Supplementary file 2 — Figure S2. Stability of the systems along MD simulations. [file FSB2-39-e70821-s005.pdf]

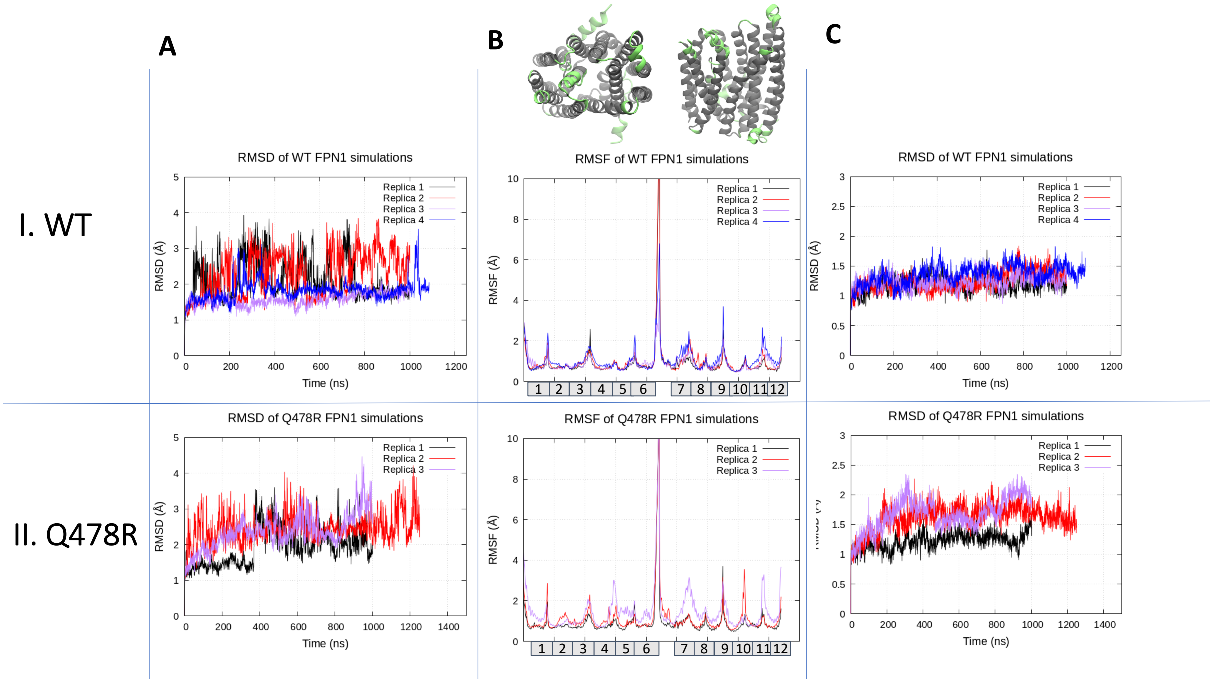

Supplementary Figure 2: Stability of the systems along MD simulations. RMSD calculated over the whole protein length (A) or the protein from which loops and the hinge region were removed (C), the mobility of which were highlighted through the analysis of RMSF values (B, green region highlighted on the 3D structure of the WT protein). I. Wild-type (WT) protein, II. Protein with the p.Gln478Arg (Q478R) variation.
