# Supplementary material for: Identification of New Key Players for Ferrous Iron Export in the Asymmetric Inner Gate of Human Ferroportin 1
Source: FASEB J. 2025 Jul 10;39(14):e70821. doi: 10.1096/fj.202500790RR (PMC12246770; doi:10.1096/fj.202500790RR)
Supplement: Supplementary file 3 — Figure S3. Membrane analysis. [file FSB2-39-e70821-s003.pdf]

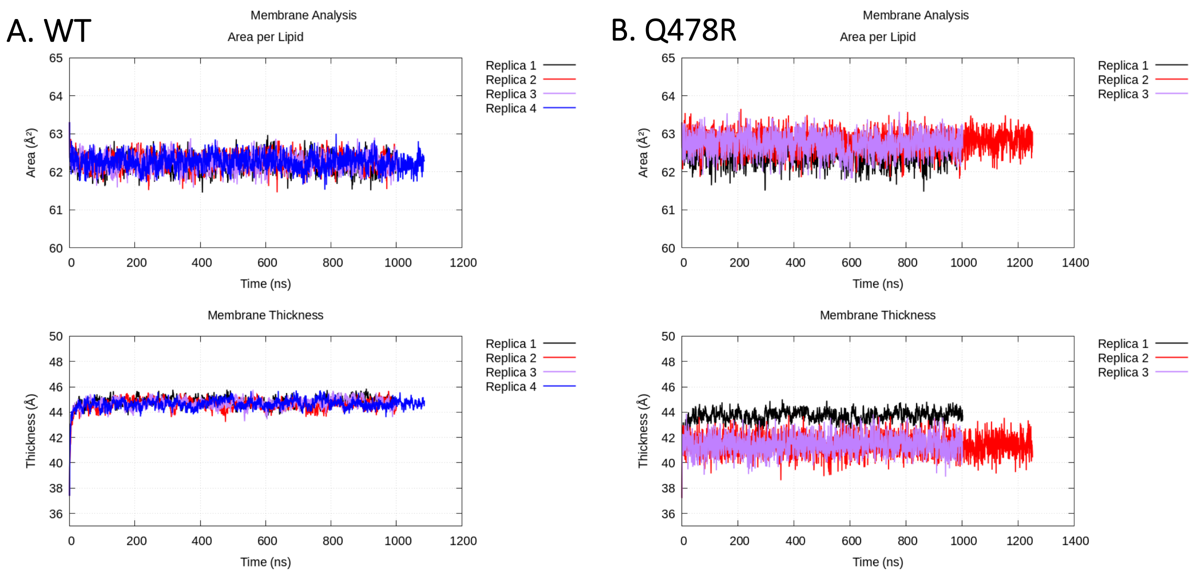

Supplementary Figure 3: Membrane analysis. The area per lipid (Å^2) and membrane thickness (Å) measured along the MD simulations. **A.** Wild-type (WT) protein, **B.** Protein with the p.Gln478Arg (Q478R) variation.
