# Supplementary material for: Identification of New Key Players for Ferrous Iron Export in the Asymmetric Inner Gate of Human Ferroportin 1
Source: FASEB J. 2025 Jul 10;39(14):e70821. doi: 10.1096/fj.202500790RR (PMC12246770; doi:10.1096/fj.202500790RR)
Supplement: Supplementary file 4 — Figure S4. Measurement of intracellular 55Fe retention versus 55Fe release in HEK293T cells not overexpressing or overexpressing wild‐type or p.Gln478Ala HsFPN1. [file FSB2-39-e70821-s007.pdf]

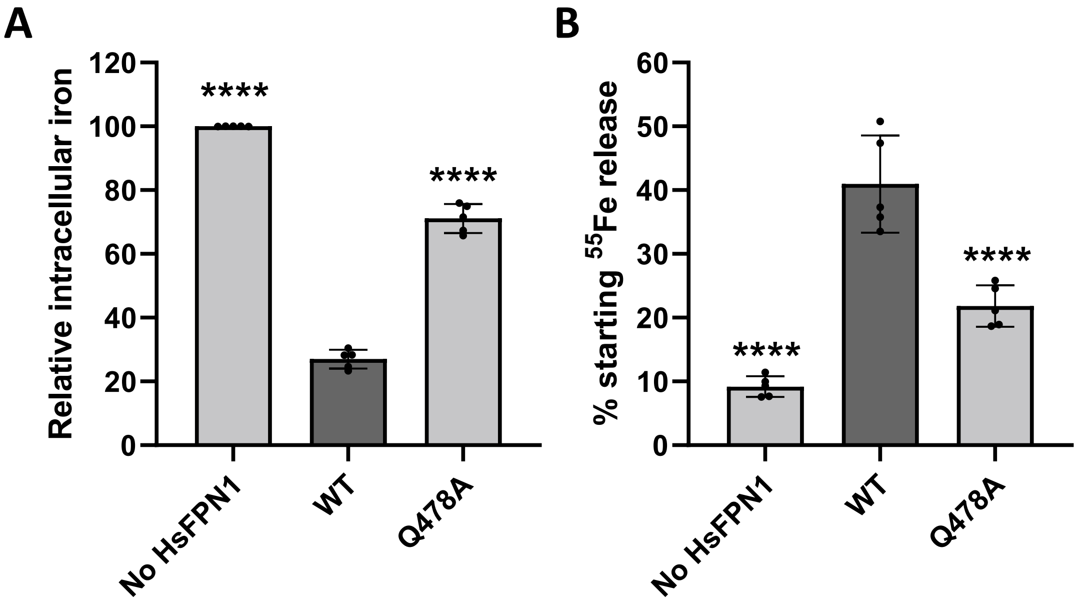

Supplementary Figure 4: Measurement of intracellular ^{55}Fe retention versus ^{55}Fe release in HEK293T cells not overexpressing or overexpressing wild-type or p.Gln478Ala HsFPN1. (A) HEK293T cells were transfected with pcDNA3.1-HsFPN1-V5-His vectors, grown for 24 h, and then fed with 20 µg/mL ^{55}Fe-transferrin for 16 h. Cells were then washed, and their ^{55}Fe content measured. Counts per minute (cpm) were normalized by total protein and β–Gal activity. Each bar represents the mean ± standard deviation of 5 independent experiments. One-way ANOVA followed by Dunnett's multiple comparisons test (control group = WT): p <0.0001 (****). (B) Transfected HEK293T cells preloaded with 20 µg/mL ^{55}Fe-transferrin for 16h were washed with PBS and cultured in serum free medium for 36h. ^{55}Fe exported into the supernatant was then counted and normalized by cellular ^{55}Fe at time zero. Each bar represents the mean ± standard deviation of 5 independent experiments. One-way ANOVA followed by Dunnett's multiple comparisons test (control group = WT): p <0.0001 (****).
