# Supplementary material for: Identification of New Key Players for Ferrous Iron Export in the Asymmetric Inner Gate of Human Ferroportin 1
Source: FASEB J. 2025 Jul 10;39(14):e70821. doi: 10.1096/fj.202500790RR (PMC12246770; doi:10.1096/fj.202500790RR)
Supplement: Supplementary file 5 — Figure S5. Sequencing electropherogram showing the SLC40A1 NM_014585.5 c.1433A>G nucleotide change. [file FSB2-39-e70821-s001.pdf]

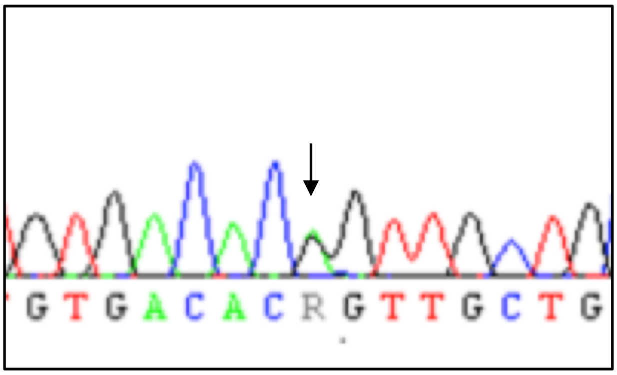

Supplementary Figure 5: Sequencing electropherogram showing the SLC40A1 NM_014585.5 c.1433A>G nucleotide change. The heterozygous position is indicated by the arrow.
