# Supplementary material for: Identification of New Key Players for Ferrous Iron Export in the Asymmetric Inner Gate of Human Ferroportin 1
Source: FASEB J. 2025 Jul 10;39(14):e70821. doi: 10.1096/fj.202500790RR (PMC12246770; doi:10.1096/fj.202500790RR)
Supplement: Supplementary file 6 — Figure S6. Effect of the p.Gln478Arg variation on cell surface expression. [file FSB2-39-e70821-s002.pdf]

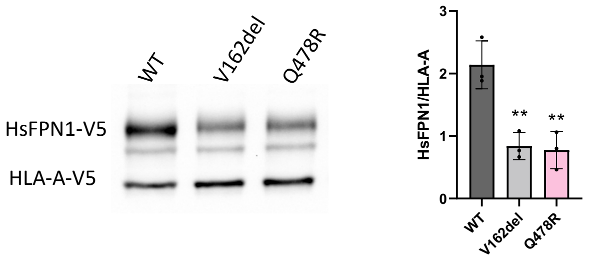

Supplementary Figure 6: Effect of the p.Gln478Arg variation on cell surface expression. HEK293T cells were transiently co-transfected with pcDNA3.1-HsFPN1-V5-His (WT or variant) and pcDNA3.1-HLA-A-V5-His vectors. Human leukocyte antigen (HLA)-A was used as a control and as a standard for normalization, being a cell-surface protein with no known role in iron metabolism. Cell-surface proteins were selectively purified 48 h after transfection and analyzed by Western blotting using a peroxidase-conjugated mouse anti-V5 antibody. Densitometric scans of HsFPN1 levels (normalized to HLA-A) are shown in the right part of the figure. The error bars represent the standard deviation of 3 independent experiments. One-way ANOVA followed by Dunnett's multiple comparisons test (control group = WT): $p < 0.01$ (**).
