# Supplementary material for: Identification of New Key Players for Ferrous Iron Export in the Asymmetric Inner Gate of Human Ferroportin 1
Source: FASEB J. 2025 Jul 10;39(14):e70821. doi: 10.1096/fj.202500790RR (PMC12246770; doi:10.1096/fj.202500790RR)
Supplement: Supplementary file 7 — Figure S7. Alpha helix irregularities in TM4 along the MD simulations of WT and Q478R HsFPN1. [file FSB2-39-e70821-s006.pdf]

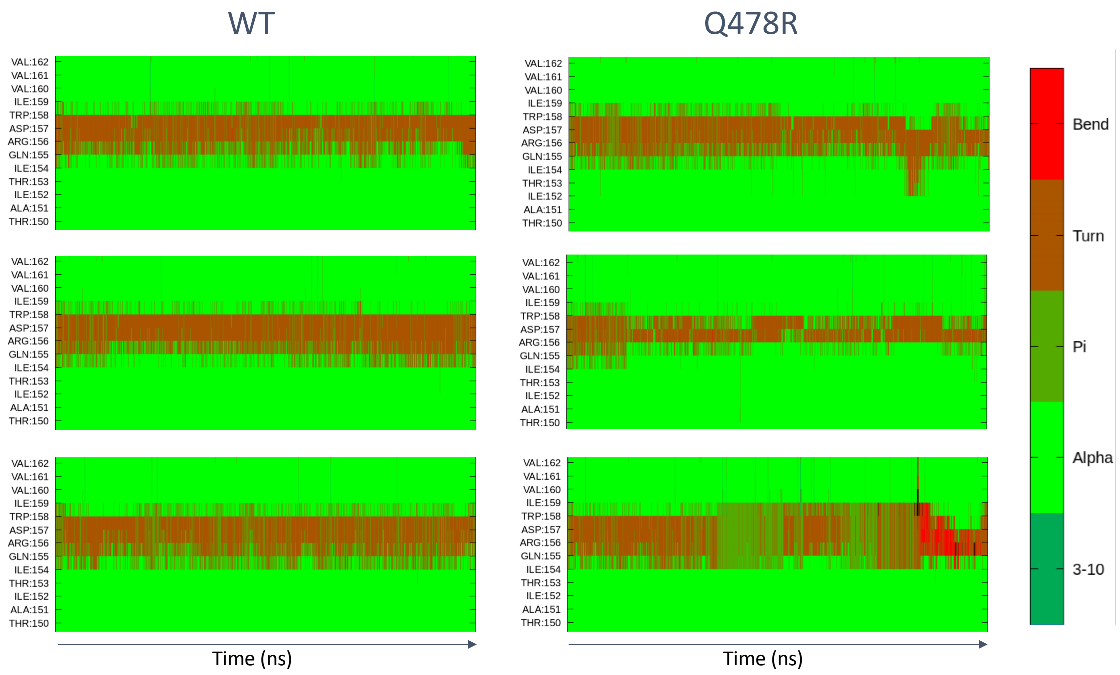

Supplementary Figure 7: Alpha helix irregularities in TM4 along the MD simulations of WT and Q478R HsFPN1. Hydrogen bond patterns were followed along the MD simulations, in order to highlight local irregularities relative to the alpha-helix pattern.
